# P-Selectin: An Unpredicted Factor for Deep Vein Thrombosis after Total Hip Arthroplasty

**DOI:** 10.1155/2014/783967

**Published:** 2014-06-25

**Authors:** Dongquan Shi, Xingquan Xu, Zhihong Xu, Takahiro Nakamura, Yong Pang, Chen Yao, Feng Wang, Dongyang Chen, Jin Dai, Qing Jiang

**Affiliations:** ^1^The Center of Diagnosis and Treatment for Joint Disease, Drum Tower Hospital Affiliated to Medical School of Nanjing University, Nanjing, Jiangsu 210008, China; ^2^Laboratory for Bone and Joint Diseases, Australian-China Joint Centre, Model Animal Research Center, Nanjing University, Nanjing, Jiangsu 210008, China; ^3^Laboratory for Mathematics, National Defense Medical College, 3-2 Namiki, Tokorozawa, Saitama 359-8513, Japan; ^4^Laboratory for Statistical Analysis, Center for Genomic Medicine, RIKEN, 4-6-1 Shirokanedai, Minato-ku, Tokyo 108-8639, Japan

## Abstract

*Introduction*. Deep vein thrombosis (DVT) is a severe complication after total hip arthroplasty (THA). It leads to acute pulmonary embolism, a life-threatening disease. P-selectin is a 140-kDa transmembrane glycoprotein. Elevated P-selectin was associated with 1.7-fold increase in the risk of venous thrombosis. *Materials and Methods*. To confirm the association, a total of 91 subjects who received primary total hip arthroplasty using lateral approach performed by one skilled orthopedic surgeon were studied. All the patients were consecutively enrolled at the Center of Diagnosis and Treatment for Joint Diseases, Drum Tower Hospital affiliated to the Medical School of Nanjing University from 2010 to 2012. All the subjects received venography 3–5 days after operation. We measured P-selectin by means of a highly sensitive sandwich ELISA technique and a commercially available test reagent set. *Results*. No significant association was detected between P-selectin and DVT (all *P*  values > 0.05). ΔsP-selectin was correlated with weight, APTT after operation, history of DVT, and diagnosis of primary disease ( *P* values were 0.03, 0.03, 0.04, and 0.02, resp.). *Conclusion*. P-selectin may not be a predicted factor for deep vein thrombosis after total hip arthroplasty.

## 1. Introduction 

Deep vein thrombosis (DVT) is a severe complication after total hip arthroplasty (THA). It leads to acute pulmonary embolism, a life-threatening disease. The prevalence of DVT after THA has ranged from 15 to 30% and 35 to 88% when prophylaxis has been used or not [1, 2]. Pulmonary embolism, most commonly originating from deep venous thrombosis of the legs, ranges from asymptomatic, incidentally discovered emboli to massive embolism causing immediate death. Acute pulmonary embolism may occur rapidly and unpredictably and may be difficult to diagnose. Treatment can reduce the risk of death, and appropriate primary prophylaxis is usually effective. Early diagnosis has been reported to reduce VTE rate and mortality [3]. There are a lot of biomarkers identified for early diagnosis of or treatment for DVT [4].

Many studies have investigated the association of the cell adhesion molecule P-selectin with blood coagulation and thrombosis [5–10]. P-selectin is partially responsible for the activation of platelets and the adhesion of certain leukocytes and platelets to the endothelium [11]. Elevated P-selectin was associated with 1.7-fold increase in the risk of venous thrombosis [12]. The plasma concentration of P-selectin is largely related to platelet activation, and the levels of P-selectin are closely associated with the risk of venous thrombosis [11]. Interaction between P-selectin and its main counterreceptor on leukocytes, P-selectin glycoprotein ligand 1 (PSGL-1), leads to neutrophil and macrophage recruitment and, along with other mediators, induces leukocytes to generate procoagulant microparticles [13].Furthermore, P-selectin triggers increased expression of tissue factor on monocytes [12] and mediates the transfer of tissue factor to platelets [14].Tissue factor, the main initiator of coagulation in vivo, causes activation of the extrinsic pathway of the coagulation cascade. A recent study revealed that P-selectin induces phosphatidylserine exposure and increases surface-dependent thrombin generation on monocytes [15].This property may represent an additional prothrombotic mechanism.

Possible roles for P-selectin in the pathogenesis of thrombosis were explored in several in vivo studies. Myers et al. [16, 17] demonstrated significantly lower thrombus weights in genetically modified animals that were deficient in P- and E-selectin compared with wild type control animals and showed that high circulating concentrations of P-selectin caused larger thrombi. Patients with venous thromboembolism (VTE) have demonstrated increased sP-selectin concentrations immediately after an acute event [18, 19] and at several months after VTE [20].

No study was reported on the association of P-selectin and acute DVT after THA. We conducted a case-control study to investigate the role of P-selectin in patients after operation of THA. Significant increase of solution P-selectin was detected after operation. However, no significant results were detected to confirm the previous reports on P-selectin as a predicate factor of DVT.

## 2. Materials and Methods 

A total of 91 subjects who received primary total hip arthroplasty using lateral approach performed by one skilled orthopedic surgeon were studied. The prostheses are from the same company. All the patients were consecutively enrolled at the Center of Diagnosis and Treatment for Joint Diseases, Drum Tower Hospital affiliated to the Medical School of Nanjing University from 2010 to 2012. No subjects dropped out during the process of the study. The study was approved by the ethical committee of the participating institutions, and informed consent was obtained from all the patients.

### 2.1. Inclusion and Exclusion Criteria

The diagnoses of patients were fracture of femoral neck, avascular necrosis of femoral head, development of dysplasia of hip, osteoarthritis, and so forth. No revision hip arthroplasty was included.

All the patients received 0.3 mL of low-molecular-weight heparin subcutaneously once daily. Prophylaxis was continued until venography was performed.

Venography was routinely performed by one doctor and randomly reviewed by at least 2 vascular surgeons. DVT was diagnosed according to the Robinov group's criterion. If DVT was detected, conventional thrombolysis treatment was to be started. If not, patients would not receive any further anticoagulation treatment.

Age, sex, DVT related history, diabetes mellitus (DM), hypertension, cancer, hormone therapy, drug, and smoking history were recorded. We measured clinical and biochemical data (weight, height, ABO blood type, PT, APTT, INR, fibrinogen, RBC, PLT, D-dimer, triglyceride, and cholesterol) and surgery-related data (blood loss, duration of surgery, EF, Hb, drainage, and symptoms of DVT). The duration of surgery, blood loss, anesthesia, and drainage all were described.

We measured sP-selectin by means of a highly sensitive sandwich ELISA technique and a commercially available test reagent set (Human sP-Selectin/CD62P ELISA reagent set; R&D Systems) according to the manufacturer's instructions. Duplicate measurements were carried out with 100 mL aliquots of plasma diluted 20-fold into sample diluent included in the ELISA reagent set and the absorbance at 450 nm with a microplate reader was measured (MR7000; Dynex Technologies/Dynatech Laboratories). We read the sP-selectin concentration from a calibration curve generated with RevelationTM software (version G 3.2) from Dynex Technologies.

## 3. Statistics

We tested the association between the DVT and the P-selectin by using Mann-Whitney test. The differences in clinical factors between the DVT statuses were compared by Mann-Whitney and Fisher's exact tests. Mann-Whitney tests, Kruskal-Wallis tests, and tests of correlation coefficient were used for detecting association between the P-selectin and other clinical factors. For all calculations of statistical analysis, the software R was used.

## 4. Results

Finally, 91 patients (64 females and 27 males) with the age of 65 ± 12.1 years old joined the study. BMI of all the patients is 24.9 ± 3.97 kg/m^2^. The incidences of DM, HP, and history of DVT are 7.7%, 35.2%, and 7.7%, respectively. 71.4% (5/7) of the patients with history of DVT recurred. 8.8% (8/91) of the patients have the habit of smoking. 42.9% (39/91), 14.3% (13/91), and 27.5% (25/91) of the patients are diagnosed with osteoarthritis (OA), developmental dysplasia of the hip (DDH), and fracture of the hip, respectively. 4 ± 1 days after THA, patients received low limb angiography routinely.

No difference was detected for all the demographic data in DVT and non-DVT groups (all *P* values >0.05) except VAS score (*P* = 0.05) (Tables 1 and 2).

The concentrations of all the patients pre-op and post-op are 2.37 ± 1.41 and 2.53 ± 1.49 ng/mL, respectively. The concentrations of DVT group pre-op and post-op are 2.14 ± 0.95 and 2.26 ± 1.04 ng/mL. And in non-DVT group pre-op and post-op, the concentrations are 2.43 ± 1.50 and 2.60 ± 1.58 ng/mL. When stratified by sex, in female, the concentrations are 2.39 ± 1.41 and 2.53 ± 1.49 ng/mL; in male, the concentrations of pre- and post-op are 2.32 ± 1.01 and 2.39 ± 1.09. No significant results were detected when we compare the difference between DVT and non-DVT groups for pre- and postsurgery and amount of change of P-selectins (all *P* values are 0.67, 0.45, and 0.98; Table 3).

### 4.1. sP-Selectin and Clinical Data

No clinical data (age, height, weight, PT, INR, APTT, etc.) was significantly correlated with sP-selectin before and after operation (Table 4). Weight, APTT after operation, history of DVT, and diagnosis of primary disease were significantly correlated with ΔsP-selectin (*P* values were 0.03, 0.03, 0.04, and 0.02, resp.). Correlation coefficients for weight, APTT after operation, and sP-selectin were 0.23 and −0.24, respectively (Tables 5, 6, and 7).

## 5. Discussion

Diagnostic strategy using P-selectin testing for DVT is still controversial. Some groups reported that the P-selectin testing to the diagnostic algorithm has the potential to make the diagnosis of DVT more convenient and economical [18, 19, 21]. However, this is the first study that reported the relationship between P-selectin and DVT after THA. This study shows that no significant results were detected to confirm the previous reports on P-selectin as a predicate factor of DVT. DVT after THA is a kind of acute condition. It is different from aetiology of DVT with abnormalities of anticoagulant and procoagulant systems. sP-selectin, as a membrane component of the platelet alpha granule and endothelial cell Weibel-Palade body, may be involved in the acute DVT but did not play a key role in its pathology.

Elevated P-selectin was detected in postoperative group. However, we did not find increased P-selectin in DVT group compared to non-DVT group. Rectenwald et al. found P-selectin levels to be significantly elevated in patients with acute DVT confirmed by duplex ultrasound [18]. Angiography is the golden standard for DVT. In our daily work, duplex ultrasound is sensitive to central thrombosis but hardly detects peripheral thrombosis. That may be the reason why we did not get similar results.

No significant difference was detected for the demographic data between DVT and non-DVT groups. Only VAS score in DVT group was significantly higher than non-DVT group. Patient who felt more pain would not like to do more rehabilitation. Then, it caused higher prevalence of DVT. Age, sex, history of DVT, blood type, BMI, PT, INR, APTT, TT, Fb, Hb, D-dimer, EF, duration of surgery, and blood loss are not risk factors in our study. There is no significant correlation between demographic data and P-selectin levels for pre- and postoperations. ΔsP-selectin had a slightly positive correlation with weight and weak negative correlation with APTT level after operation. As platelet is activated in the pathogenesis of obesity and vascular disease, it is possible to have higher P-selectin level in DVT patients. smoke is a critical responsibility for DVT in the previous reports [21]. However, we did not find it in the present study, either for sex.

## 6. Conclusion

P-selectin may not be a predicted factor for deep vein thrombosis after total hip arthroplasty.

## Figures and Tables

**Table 1:** DVT versus the continuous covariates.

Subjects	DVT = 1		DVT = 2		*P* value
	Mean	S.D.	Mean	S.D.	
Age	65.12	12.47	66.50	10.57	0.85
Height	161.10	8.21	160.76	8.51	0.87
Weight	64.03	10.92	65.91	9.34	0.77
PT	11.65	0.72	11.54	0.65	0.57
INR	1.41	3.35	1.01	0.06	0.53
APTT	25.54	5.43	26.27	3.48	0.59
TT	17.99	2.24	18.22	2.17	0.59
Fb	3.23	0.72	3.20	0.72	0.93
Hb	127.11	15.77	128.50	14.51	0.65
PLT (pre)	188.15	59.97	184.39	49.66	0.96
D-dimer	0.29	0.55	0.20	0.29	0.33
EF	59.33	10.89	60.25	2.67	0.25
ESR	30.62	14.10	32.35	11.80	0.64
OP time	117.64	33.61	108.82	30.03	0.39
Blood lost	522.60	319.73	520.59	351.36	0.84
VAS	3.54	1.16	4.12	1.22	0.05
RBC post-op	3.53	0.50	3.57	0.51	0.89
Hb post-op	106.35	13.50	104.15	27.98	0.67
PT post-op	155.53	58.15	149.96	62.26	0.96
PT post-op	12.11	0.84	11.92	0.71	0.46
INR post-op	1.06	0.07	4.43	14.37	0.72
APTT post-op	27.19	5.29	28.49	9.05	0.35
TT post-op	15.71	1.75	21.31	22.96	0.34
Fb post-op	4.12	0.68	9.34	21.14	0.09

*P* values were obtained by using Mann-Whitney test.

**Table 2:** DVT versus the discrete covariates.

Subjects	*P* value
Sex	0.39
DM	0.35
HP	0.79
Cardio	0.58
History of DVT	0.35
History of surgery	0.30
Smoke	1.00
Diagnosis	0.43
Blood type	0.43
Edema	0.36

*P* values were obtained by using Fisher's exact test.

**Table 3:** DVT versus p-selectin.

Subjects	DVT = 1		DVT = 2		*P* value
	Mean	S.D.	Mean	S.D.	
P-selectin (pre)	2.43	1.50	2.14	0.95	0.67
P-selectin (post)	2.60	1.58	2.26	1.04	0.45
Amount of change of p-selectins	−0.17	0.57	−0.12	0.33	0.98

*P* values were obtained by using Mann-Whitney test.

**Table 4:** P-selectin versus the continuous covariates.

Subjects	P-selectin (pre)		P-selectin (post)		Amount of change of P-selectins	
	Correlation coefficients	*P* value	Correlation coefficients	*P* value	Correlation coefficients	*P* value
Age	0.15	0.17	0.18	0.08	−0.13	0.23
Height	−0.10	0.37	−0.10	0.35	0.03	0.81
Weight	0.01	0.93	−0.07	0.50	0.23	0.03
PT	−0.13	0.23	−0.14	0.17	0.07	0.53
INR	0.06	0.59	0.10	0.37	−0.12	0.25
APTT	−0.01	0.89	0.05	0.66	−0.17	0.11
TT	0.05	0.65	0.03	0.75	0.03	0.75
Fb	0.06	0.56	0.02	0.88	0.12	0.25
Hb	−0.12	0.26	−0.13	0.21	0.06	0.58
PLT (pre)	0.13	0.21	0.08	0.43	0.12	0.26
D-dimer	−0.12	0.28	−0.04	0.70	−0.20	0.07
EF	−0.01	0.94	−0.04	0.70	0.10	0.36
ESR	0.03	0.81	0.03	0.79	−0.02	0.88
OP time	0.10	0.34	0.08	0.45	0.05	0.66
Blood lost	0.13	0.22	0.10	0.36	0.08	0.48
VAS	−0.12	0.25	−0.07	0.51	−0.13	0.23
RBC post-op	−0.09	0.43	−0.05	0.63	−0.07	0.53
Hb post-op	−0.03	0.77	0.04	0.72	−0.17	0.12
PT post-op	0.06	0.60	0.05	0.68	0.02	0.84
PT post-op	−0.15	0.15	−0.20	0.06	0.15	0.16
INR post-op	0.03	0.78	0.00	1.00	0.08	0.45
APTT post-op	0.02	0.83	0.10	0.33	−0.24	0.03
TT post-op	0.06	0.59	0.01	0.89	0.11	0.29
Fb post-op	0.02	0.83	0.00	0.98	0.07	0.51

**Table 5:** P-selectin (pre) versus the other discrete covariates.

Subjects	Categories										*P* value	Categorization
	0		1		2		3		4	
	Mean	S.D.	Mean	S.D.	Mean	S.D.	Mean	S.D.	Mean	S.D.
DVT	2.43	1.50	2.14	0.95							0.67	Normal = 0, DVT = 1
Sex	2.39	1.55	2.32	1.01							0.72	Female = 0, male = 1
DM	2.41	1.44	1.95	0.94							0.40	
HP	2.31	1.09	2.49	1.87							0.58	
Cardio	2.41	1.43	1.65	0.65							0.13	
History of DVT	2.33	1.34	2.77	2.08							0.78	
History of surgery	2.20	1.14	2.57	1.69							0.18	
Smoke	2.39	1.44	2.17	1.02							0.82	
Diagnosis	2.57	1.77	1.77	0.93	2.32	1.02	2.81	1.67	2.29	0.80	0.33	
Blood type	2.15	0.87	2.31	1.16	2.95	2.53	2.43	1.62			0.89	A = 0, B = 1, AB = 2, O = 3
Edema	2.26	1.21	2.55	1.69	2.37	1.60	1.03				0.39	Light = 1, middle = 2, severe = 3

**Table 6:** P-selectin (post) versus the other discrete covariates.

Subjects	Categories										*P* value	Categorization
	0		1		2		3		4	
	Mean	S.D.	Mean	S.D.	Mean	S.D.	Mean	S.D.	Mean	S.D.
DVT	2.60	1.58	2.26	1.04							0.45	Normal = 0, DVT = 1
Sex	2.59	1.64	2.39	1.09							0.89	Female = 0, male = 1
DM	2.59	1.51	1.91	1.26							0.19	
HP	2.50	1.19	2.59	1.96							0.52	
Cardio	2.58	1.51	1.65	0.89							0.11	
Histor of DVT	2.53	1.47	2.58	1.85							0.67	
History of surgery	2.35	1.30	2.72	1.71							0.35	
Smoke	2.56	1.53	2.23	1.07							0.67	
Diagnosis	2.58	1.79	1.77	1.03	2.78	1.18	3.00	1.77	2.47	1.17	0.12	OA = 0, AVN = 1, fracture = 2, DDH = 3, others = 4
Blood type	2.25	1.03	2.51	1.32	2.89	2.09	2.68	1.83			0.81	A = 0, B = 1, AB = 2, O = 3
Edema	2.45	1.29	2.68	1.84	2.49	1.40	0.78				0.50	Light = 1, middle = 2, severe = 3

Mann-Whitney test and Kruskal-Wallis test were used for obtaining *P* values.

**Table 7:** Amount of change of P-selectins versus the other discrete covariates.

Subjects	Categories										*P* value	Categorization
	0		1		2		3		4	
	Mean	S.D.	Mean	S.D.	Mean	S.D.	Mean	S.D.	Mean	S.D.
DVT	−0.17	0.57	−0.12	0.33							0.98	Normal = 0, DVT = 1
Sex	−0.20	0.57	−0.07	0.42							0.39	Female = 0, male = 1
DM	−0.18	0.54	0.04	0.39							0.12	
HP	−0.19	0.56	−0.10	0.46							0.53	
Cardio	−0.17	0.54	0.00	0.28							0.44	
History of DVT	−0.19	0.52	0.20	0.47							0.04	
History of surgery	−0.15	0.57	−0.15	0.48							0.29	
Smoke	−0.17	0.52	−0.06	0.59							0.58	
Diagnosis	0.00	0.44	−0.01	0.32	−0.46	0.66	−0.19	0.20	−0.18	0.55	0.02	OA = 0, AVN = 1, fracture = 2, DDH = 3, others = 4
Blood type	−0.10	0.51	−0.20	0.45	0.06	0.67	−0.25	0.55			0.65	A = 0, B = 1, AB = 2, O = 3
Edema	−0.18	0.58	−0.13	0.41	−0.13	0.67	0.25				0.62	Light = 1, middle = 2, severe = 3

Mann-Whitney test and Kruskal-Wallis test were used for obtaining *P* values.
